# Ex Vivo Activation of Red Blood Cell Senescence by Plasma from Sickle-Cell Disease Patients: Correlation between Markers and Adhesion Consequences during Acute Disease Events

**DOI:** 10.3390/biom11070963

**Published:** 2021-06-30

**Authors:** Philippe Chadebech, Gwellaouen Bodivit, Gaétana Di Liberto, Alicia Jouard, Corinne Vasseur, France Pirenne, Pablo Bartolucci

**Affiliations:** 1 Etablissement Français du Sang (EFS) Île-de-France, Hôpital Henri-Mondor, F-94000 Créteil, France; gwellaouen.bodivit@efs.sante.fr (G.B.); alicia.jouard@efs.sante.fr (A.J.); france.pirenne@efs.sante.fr (F.P.); 2 INSERM U955 Équipe Pirenne “Transfusion et Maladies du Globule Rouge” et Institut Mondor de Recherche Biomédicale (IMRB), F-94000 Créteil, France; gaetanadiliberto@yahoo.fr (G.D.L.); corinne.vasseur@inserm.fr (C.V.); pablo.bartolucci@hmn.aphp.fr (P.B.); 3 Laboratoire d’Excellence GR-Ex, F-75739 Paris, France; 4 Université Paris Est Créteil, INSERM, IMRB, F-94010 Créteil, France; 5 Centre de Référence pour la Drépanocytose, Unité des Maladies Génétiques du Globule Rouge, Hôpital Henri-Mondor, F-94010 Créteil, France

**Keywords:** sickle-cell disease, transfusion, RBC’ senescence, phosphatidylserine, hydration

## Abstract

BACKGROUND: Blood transfusion remains a key treatment for managing occlusive episodes and painful crises in sickle-cell disease (SCD). In that clinical context, red blood cells (RBCs) from donors and transfused to patients, may be affected by plasma components in the recipients’ blood. Senescence lesion markers appear on the red cells after transfusion, shortening the RBC lifespan in circulation. In the specific context of SCD, senescence signals can also trigger the occlusive painful events, typical of the disease. This work follows through our previous data that described a RBC senescence process, rapidly detected after challenge with SCD pathological plasmas. In this clinical context, we wanted here to further explore the characteristics and physiologic consequences of AA RBC lesions associated with senescence, as lesions caused by RBCs after transfusion may have adverse consequences for SCD patients. METHODS: Plasma samples from SCD patients, with acute symptoms (*n* = 20) or steady-state disease (*n* = 34) were co-incubated with donor AA RBCs from blood units for 24 to 48 h. Specific markers signing RBC senescence were quantified after the incubation with SCD plasma samples. The physiologic in-flow adhesion was investigated on senescent RBCs, an in vitro technic into biochips that mimic adherence of RBCs during the occlusive events of SCD. RESULTS: Senescence markers on AA RBCs, together with their in-flow adhesion to the plasma-bridging protein thrombospondin, were associated with the clinical status of the SCD patients from whom plasma was obtained. In these experiments, the highest values were obtained for SCD acute plasma samples. Adhesion of senescent RBCs into biochips, which is not reversed by a pre-treatment with recombinant Annexin V, can be reproduced with the use of chemical agents acting on RBC membrane channels that regulate either Ca^2+^ entry or modulating RBC hydration. CONCLUSION: We found that markers on red cells are correlated, and that the senescence induced by SCD plasma provokes the adhesion of RBCs to the vessel wall protein thrombospondin. In-flow adhesion of senescent red cells after plasma co-incubations can be reproduced with the use of modulators of RBC membrane channels; activating the Piezo1 Ca^2+^ mechanosensitive channel provokes RBC adhesion of normal (non-senescent) RBCs, while blocking the Ca^2+^-dependent K^+^ Gardos channel, can reverse it. Clinically modulating the RBC adhesion to vascular wall proteins might be a promising avenue for the treatment of painful occlusive events in SCD.

## 1. Introduction

Blood transfusion is part of the therapeutic arsenal in various diseases, for increasing circulating hemoglobin (Hb) levels and improving oxygen delivery to tissues [1]. To assume this function, normal hemoglobin (Hb)A-carrying red blood cells (RBCs) stored in blood units over a 42-day period in banks [2,3] must keep optimal capacities: their ability to off-load oxygen to the tissues, to change their shape in order to easily circulate throughout the microvascular network and to avoid morphological modifications. For this purpose, donor AA RBCs are supplemented with an additive solution during the preparation of packed blood units, to maintain quality over storage [4,5]. Nevertheless, the process of RBC aging naturally occurring in the circulation also occurs during storage in blood bags. This is due to the rapid accumulation of irreversible ‘storage lesions’, which may reduce the lifespan of HbA-carrying RBCs after transfusion into patients [2,3,6,7,8]. Storage lesions display features common to both physiological aging in circulation and accelerated RBC “death” by senescence, triggered by adverse compounds or environments [9,10,11,12]. Modifications include an increased RBC rigidity, the emergence of neoantigenic domains, or the externalization of anionic phospholipids [13]; the Band 3 membrane protein also aggregates, becoming the target of senescence autoantibodies [14]. A glycophorin-C desialylation occurring during ageing, that led to increased adhesiveness of red cells through a Lutheran/basal cell adhesion molecule (Lu/BCAM) ‒ laminin-α5 interaction, is also documented [15].

The intracellular Ca^2+^ increase (i.e., calcium influx) occurring in altered or physiologically aged RBCs seems to be a central event leading to RBC “eryptotic” senescence or ageing. Calcium increase into red cells modifies the physical properties of RBCs by regulating cell volume [16,17]; this activates the calcium-dependent K^+^ Gardos/KCa3.1 channels, modulating RBC hydration and leading to a decrease in size [10,17,18]. The scrambling of phospholipids, which is also a calcium-sensitive mechanism, leads to phosphatidylserine (PS)-exposure on the outer membrane, a key signal for RBC elimination. PS-exposure on RBCs may then favor adhesion to endothelial cells via the CXC chemokine ligand 16 (CXCL16) [19,20] or phagocytosis by PS receptor-bearing cells [21,22]. 

With the use of stored RBC units selected on the basis of their relative age (“fresh” (5 to 8 days) versus “old” (≥38 days) units), we previously demonstrated that SCD plasmas can adversely affect RBCs from AA donor units in co-incubations at +37 °C [23] with the “old” RBCs more affected by pathological environments from acute patients [9,12,23]. Plasma samples from such patients in occlusive episodes or hemolytic reactions may contain oxidative agents or secreted factors potentially harmful to RBC membranes, with lesions occurring during the storage, not detectable on the RBCs in donor units, rapidly emerging in the bloodstream of the recipient after transfusion [24,25,26].

The transfusion of blood units is often required in patients with homozygous (SS) sickle-cell disease (SCD), a monogenic red blood cell disorder that provokes the formation of an abnormal polymerizing hemoglobin, named HbS [1,9,26]. In addition to their propensity to sickle, rigidify and hemolyze, the SS RBCs also adhere to the vessel wall proteins, and among them thrombospondin (TSP), a secreted glycoprotein playing a role into the extracellular matrix of endothelial cells (ECs). Abnormal interactions between RBCs and endothelium into patients play a critical role in the occurrence of the painful episodes or vaso-occlusive events in SCD [27,28]. In that disease context, the transfusion of donor blood units, that brings normal HbA RBCs in circulation, is able to treat or prevent these painful crisis [9,23,26] by reducing the percentage of RBCs with abnormal HbS. 

Another characteristic and specificity of SCD patients is the detection of abnormally dense RBCs (density  >1.11) in circulation, that comprise a substantial number of irreversibly sickled cells with HbS polymer. They have high mean corpuscular Hb concentrations due to dehydration [28,29]. In one way, dense SS RBCs detected in SCD share some characteristics with senescent AA RBCs from blood units obtained ex vivo. The dense RBC % is also a stable individual biological characteristic in adult SCD patients [29,30]. Bartolucci and colleagues from our team recently showed that dehydrated dense RBCs have the ability to adhere to vessel wall proteins, with the mechanism responsible for this adhesion still unknown [28]. The question arises, therefore, whether senescent RBCs can share similar adhesion properties with dense RBCs, also participating in vessel occlusions in SCD.

In this work, we wanted to shed additional light on red cell senescence parameters, previously detected in our team after challenge with plasmas and further explored the characteristics and behavior of senescent RBCs due to contact with sickle-cell plasmas, as lesions caused to donor-transfused AA RBCs may have adverse consequences in the transfusion context of patients with SCD. Notably, we investigated the correlations existing between the markers appearing on senescent red cells, which have never been shown before, and the possible contribution of senescent AA RBCs to the adverse consequences of RBC adherence that occur in sickle painful crisis. 

## 2. Study Design and Methods

Full details regarding the recruitment of patients and controls, the selection of RBC units, and the methods used are provided in the Supplementary Materials. The medical ethics committee of Henri-Mondor Hospital (Créteil) approved this study (CPP no.11-047). Anonymized data from the patients were analyzed in accordance with the Declaration of Helsinki. 

Processing the RBC units from whole blood of (AA) donors was carried out at EFS Preparation (Rungis, France) according to the European guidelines [4]; the RBC concentrates were stored at +4–6 °C in accordance with requirements for therapeutic blood units. For the present study, they were drawn aside from therapeutic delivery circuit on the unique criteria of their too small volume (<300 mL) [12] and were received at our laboratory on day 3 post blood collection. The RBCs were sampled from the blood units from 7 to 15 days of storage. Before co-incubations with plasma samples, the RBCs were washed twice in 0.9% NaCl and packed by centrifugation at 2880× *g* for 10 min at room temperature. 

Plasma samples were obtained from 54 randomly selected adult patients with a definitive diagnosis of SCD (homozygous Hb SS; ≥18 years; 21 male/33 female; median age: 32.5 ± 7.9 years) from Henri-Mondor Hospital (Créteil, France). Patients were followed for routine explorations outside of painful episodes (“Steady state”; *n* = 31) or during hospitalization for acute symptoms (“Acute phase”; *n* = 23). The selected SCD plasma were thawed and incubated with RBCs at an 8% Hct drawn from stored blood units, for 24 or 48 h at +37 °C, as previously described [9] to mimic the temperature of plasma contact in the bloodstream after transfusion. A mixture of RBCs pooled from 3 to 5 individual AA donors’ blood units was made to minimize individual variability between the units. In previous investigations already published [12,23], we verified that the co-incubation of normal AA RBCs from units, either without any plasma contact or with contact HD plasmas, for 24 or 48 h, only slightly induce the appearance of the various senescence markers [12]. Incubations between RBCs and sickle plasmas were carried out on different days to avoid the batch effect. Following the incubation of AA RBCs with the SCD plasma samples, markers of senescence were quantified by flow cytometry, with assessments of the PS exposure based on Annexin V-FITC staining, the RBC size based on the FSC Geo Mean parameter and the calcium influx based on the Fluo3/AM loading, as previously described [12,23]. Analyses were performed on a Canto2 flow cytometer (BD) with the Diva software. 

The adhesion of RBCs to thrombospondin was measured under physiological in-flow conditions, as previously described [28]. We used thrombospondin from human platelets (TSP; 10 μg/mL) immobilized into Vena8 microslides from Cellix Ltd. The senescent AA RBCs obtained after a 48 h incubation with the various plasma samples were resuspended at 0.5% hematocrit in HBSS/BSA and perfused through the microslide with VenaFlux Assay 2.3 analysis software, on a fully automated Z1 microscope (Carl Zeiss) at a shear stress of 0.5 Dyn/cm^2^ for 5 min at +20 °C. Washouts were then performed with Hank’s buffer, at increasing shear stresses from 0.5, then to 2 and finally to 5 Dyn/cm^2^ at +20 °C, for 5 min each, and adherent cells were counted. 

Where indicated, the addition of recombinant Annexin V (5 and 50 µg) to diluted AA RBCs (0.5% Hct) was carried out for a 10 min incubation at +4 °C before processing the in-flow adhesion assays. Where indicated, fresh native RBCs from blood units were treated with the Piezo1/TRPC5 channel agonist Yoda1 (50 mM) for 30 min at +37 °C, with or without the Gardos/KCa3.1 inhibitors Senicapoc or TRAM-34 (10 mM each) as pretreatments for 10 min at +37 °C before Yoda-1 addition. At the end of these treatments, diluted AA RBCs (0.5% Hct in HBSS /BSA) were processed the flowing adhesion assays. 

Statistical analysis was performed with Kruskal–Wallis, Mann–Whitney U, or two-tailed Student’s *t* test (Wilcoxon signed-rank tests), as appropriate, with the Prism software (GraphPad Inc). Values of *p* < 0.05 were considered significant. * *p* < 0.05, ** *p* < 0.01, *** *p* < 0.001, **** *p* < 0.0001. Linear regressions were made to test the correlations between the senescence markers for the patients’ plasma, tested for whom we collected results for the three different markers (*n* = 20) using the Stata software (StataCorp).

## 3. Results

### 3.1. Exposure of Donor AA RBCs from Blood Units to Pathological Plasma from SCD Patients Leads to RBC Senescence Correlated with the Patients’ Clinical Status

First, we confirmed here the previous findings obtained from our team and from other groups [10,19] showing that incubation of normal (AA) RBCs freshly drawn from blood units with pathological plasma samples, from SCD patients notably [22] can rapidly generate “eryptotic” AA RBCs showing senescence markers. Among them, the phospholipid phosphatidylserine (PS) is found exposed at their membrane surface. We already verified that the co-incubation of normal AA RBCs from units, either without any plasma contact or with contact HD plasmas, for 24 or 48 h, only slightly lead to the appearance of the various senescence markers [12,23] and were not described again in the present study.

We found that the PS-positive RBC percentage (PS+ RBC%) is dependent and correlated with the time of in vitro contact at +37 °C between AA RBCs from units and sickle plasmas (see: Supplementary Figure S1): the PS+ RBC% was relatively low after T 24 h, whatever the clinical condition of the patient considered, by comparison to the T 48 h time period (*p* < 0.0001; Wilcoxon test). 

**Figure 1:**
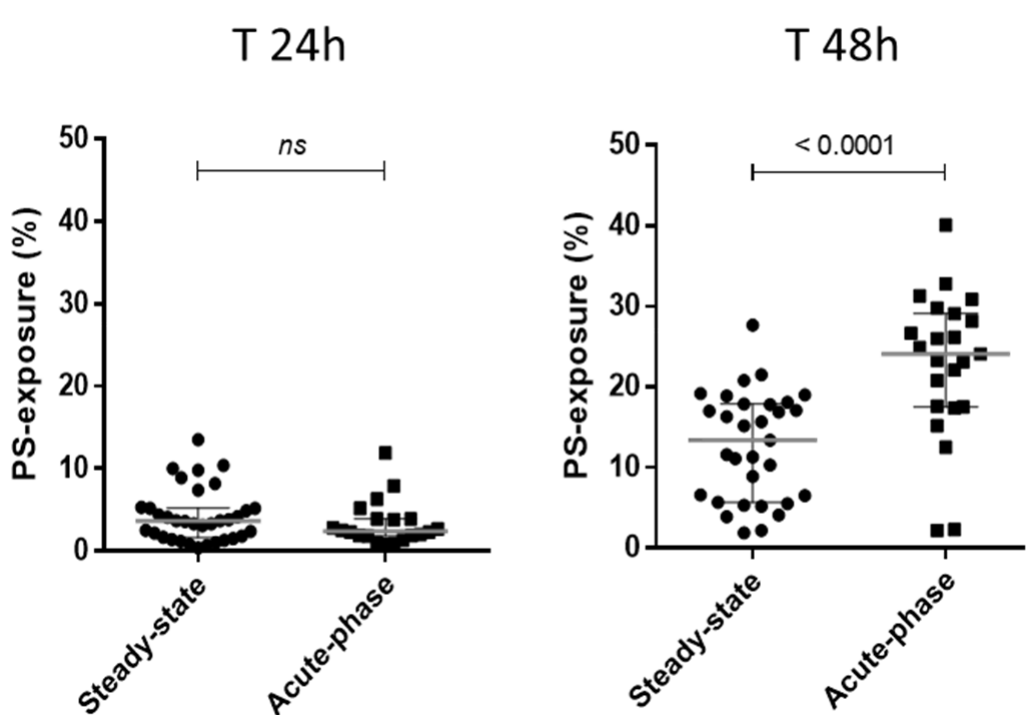
Phosphatidylserine-exposure on RBCs after co-incubations with sickle-cell disease patient plasmas. The results obtained concerning PS-detection on AA RBCs after the 24-h (**T 24 h**) and 48-h (**T 48 h**) incubations at +37 °C with SCD plasma samples, are represented considering the clinical condition of the patients (“steady-state”, *n* = 31 versus “acute-phase”; *n* = 23). The PS-exposure results obtained were 4.26 ± 3.2 % and 3.35 ± 2.7 %, respectively; at T 24 h (*p* = 0.2999; left plots), 12.66 ± 6.7 % and 22.8 ± 9.03 %, respectively; at T 48 h (*p* < 0.0001; right plots). *P* values indicated on the graphs represents results of Mann–Whitney U tests (see also: Supplementary Figures S1 and S2, Table S1).

The SCD patients were then subdivided as a function of the severity of patient clinical status, into “acute-phase” plasmas (corresponding to samples from painful occlusive crisis patients, with or without ACS) and basal “steady-state” plasmas (collected from patients out of the context of painful episodes or transfusions) (Figure 1). A weak PS+ RBC population was detected after a 24-h incubation, with no difference between the two plasma sample categories (left plot, *p* = 0.2999). After 48 h, the PS+ RBC population was significantly larger for plasma samples collected in painful conditions (‘Acute-phase’; right plot) than in those collected in basal “steady-state” conditions (*p* < 0.0001) (see also: Supplementary Figure S1). Therefore, we only used the T 48 h samples of AA RBCs incubation with plasmas for the following adhesion tests. 

Then, a two-way ANOVA factorial analysis revealed that the clinical state of the patients’ plasmas tested (“steady state” versus “acute phase”) had a highly significant effect (*p* < 0001) on PS-exposure quantification. The duration of the RBC/plasma contact (T 24 h and T 48 h) had a less but also significant effect (*p* = 0001) and a significant interaction between the two variables is observed (*p* < 0001) (see also: Supplementary Figure S2 and Table S1). 

### 3.2. The Levels of Senescence Markers on AA RBCs from Blood Units Are Correlated after a 48 h Incubation Period with Plasma from SCD Patients

**Figure 2:**
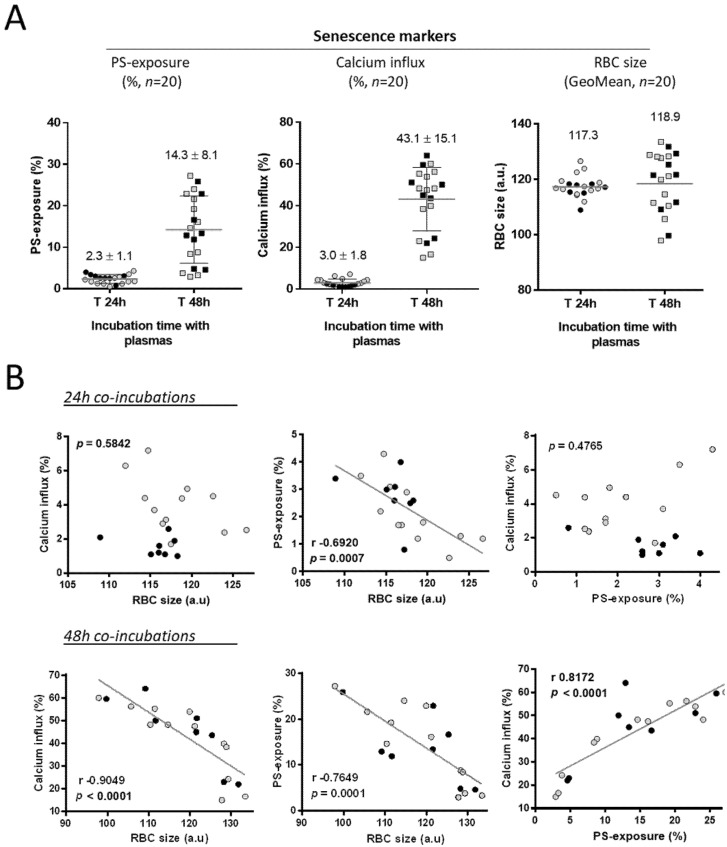
Markers of senescence detected on RBCs after co-incubations with SCD patient plasmas and correlations between changes in senescence markers for senescence on AA RBCs after 24-h (upper panel) and 48-h (lower panel) co-incubations. (**A**) AA RBCs from blood units and the patient plasmas (*n* = 20) were used for co-incubations together at +37 °C for T 24 h and T 48 h. The senescence markers were the following: the PS-exposure on red cells (Annexin V FITC positive cells, %), the level of calcium influx into red cells (Fluo3/AM staining; %) and the reduction in RBC size attested by the FSC GeoMean parameter (arbitrary units), all by flow cytometry. We represented the results for the 20 individual SCD plasma samples for whom we had collected data for the three senescence markers. The mean values obtained (% ± SD for PS-exposure and calcium influx) or the GeoMean (for the RBC size) are represented on each graph. (**B**) The plots represent the Pearson correlations (r; *p*) after linear regression of the different parameter combinations, by using the data shown in Figure 1: the relative RBC size (FSC GeoMean parameter) and the calcium influx into red cells (%; Fluo3/AM parameter), the RBC size and the PS-exposure (%) (Annexin V-FITC positive staining) the PS-exposure (%) and the calcium influx into red cells (%). Each dot represents the plasma from an individual SCD patient; using the same data used in Figure 1, and a distinction is made between samples obtained from SCD patients in a basal steady state (light-grey circles) compared to SCD patients in a painful acute-phase (black circles) of the disease. Linear regressions and the correlations possibly existing between the senescence markers were tested using the Stata software.

After these first results concerning the PS-exposure of senescent AA RBCs after plasma challenge, at 24 and 48 h co-incubations (Figure 1; *n* = 54 plasmas), we needed to show the results for the three senescence markers in parallel. For this, we detailed in three graphs the results obtained for 20 individual SCD plasma samples for whom we had collected data, at T 24 h and T 48 h co-incubation, for the three RBC markers: PS-exposure on red cells, calcium influx into red cells and reduction in RBC size (Figure 2A). As the clinical context of the SCD patients could have an importance in the appearance of the senescence markers, we individualized the data collected for plasma from the patients in a basal steady state (grey dots) in parallel opposition to plasma from the patients in an acute phase of the disease (black dots). The high increase in PS-exposure on senescent RBCs at T 48 h (left graph) is coincident with a large detection of calcium influx into red cells (center graph). On this subpopulation of 20 plasmas co-incubated with native AA RBCs from units, we didn’t observe any significant decrease in the RBC size (FSC Geo Mean; right graph). As expected, the results obtained at T 24 h for PS-exposure and Ca^2+^ influx positive red cells (%) were near zero for both markers. 

Then, we wanted to explore the correlation possibly existing between the principal markers attesting RBC senescence, by investigating linear regressions and performing Pearson r correlation analyses (Figure 2B) (see: Supplementary Materials for more details) to determine the relationship between two variables. As in Figure 2A, we individualized the data collected for plasma from the patients in a basal steady state (grey dots) in parallel to plasma in an acute phase (black dots). 

Following co-incubations for 24 h with the plasma samples (Figure 2B, upper panel), we found no correlation was found between the RBC decrease in size and calcium influx into red blood cells (*p* = 0.5842). Similarly, no correlation was found between PS-exposure (Annexin V-FITC positive staining) and calcium influx (*p* = 0.4765). Only the changes quantified between PS-exposure and RBC size were found to be inversely correlated (r: −0.6920; *p* = 0.0007). Following the incubation of the AA RBCs for 48 h, all the markers of RBC senescence were found to be well correlated (Figure 2B, lower panel). 

### 3.3. Senescence of AA RBCs Induced by SCD Plasma Samples Leads to in-Flow Adhesion to Thrombospondin

**Figure 3:**
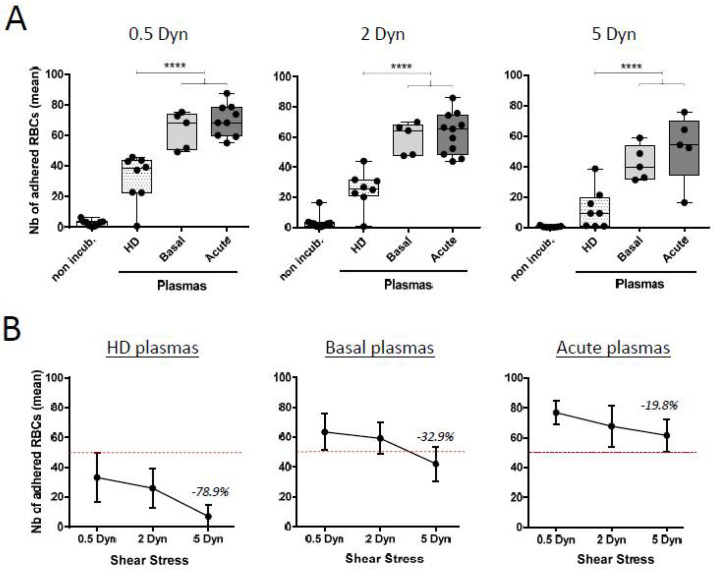
Accelerated senescence of AA RBCs after co-incubations with SCD plasmas induce adhesion to thrombospondin. (**A**) The flowing adhesion of donor AA RBCs from blood units was tested after co-incubations with plasmas from SCD patients (the respective “basal” and “acute” clinical conditions) or from healthy donors (“HD”), with TSP immobilized in microslides. The RBCs were perfused through the microslide using the VenaFlux software. Washouts were made at increasing physiologic shear stresses (0.5, 2 and 5 Dyn/cm^2^) conditions for 5 min each. Adherent red cells were counted in five representative areas. The mean values obtained for the adhesion of non-incubated AA RBCs for the respective shear stress 0.5, 2 and 5 Dyn were 2.6 ± 1.9, 3.5 ± 4.7 and 0.4 ± 0.5. The mean values obtained for adhered RBCs incubated with “HD” plasmas were 31.9 ± 15.5, 25.2 ± 12.3 and 12.1 ± 13, respectively. Results obtained for adhered RBCs incubated with “basal” plasmas were 63.4 ± 12.1, 59.2 ± 10.5 and 42.2 ± 11.5, respectively; and for adhered RBCs incubated with “acute” plasmas: 69.8 ± 10.6, 62.2 ± 16.6 and 52.6 ± 22.3, respectively. (**** *p* < 0.0001; one way ANOVA) (**B**) The strength of RBC adhesion to TSP under physiologic flow conditions in microslides was quantified using the mean results of RBC adhesion obtained in (A). The mean results for adhered RBCs after contact with “HD” plasmas were the following: 33.2 ± 16, 25.9 ± 13 and 7.1 ± 8 for the respective shear stress values 0.5, 2 and 5 Dyn. Results for adhered RBCs after contact with “basal” plasmas were the following: 63.4 ± 12 (0.5 Dyn), 59.2 ± 10 (2 Dyn) and 42.2 ± 12 (5 Dyn). The mean results for adhered RBCs after contact with “acute” plasmas were 76.9 ± 8 (0.5 Dyn), 67.8 ± 14 (2 Dyn) and 61.6 ± 11 (5 Dyn). A dotted line is indicated on the three graphs, arbitrary positioned upper to the maximal values for 0.5 Dyn for “HD” plasmas (mean + upper SD) to demonstrate the differences in the adhesion results for the different categories of plasmas. Numbers in percentage indicate on the graphs the respective changes obtained for each category of plasma between the 0.5 Dyn and the 5 Dyn relative shear stress applied in the test. Decreases in the number of adhered RBCs to TSP were –78.9%, –32.9% and –19.8%, respectively. Results represent the mean of five to seven independent experiments.

Abnormal interactions between RBCs and endothelial cells can play a critical role in the occurrence of the painful vaso-occlusive events associated with SCD. Then, the adhesion of AA RBCs to TSP was investigated into physiological assays under flowing conditions, as previously described [26], using some of the plasma samples from selected SCD patients in a basal steady-state (“basal”; *n* = 5) or experiencing a painful acute-phase episode (“acute”; *n* = 11) at the time of sample collection. As a control, plasma samples from healthy adult donors (“HD”; *n* = 8) were also tested (Figure 3A). We showed that AA RBCs incubated with normal “HD” or pathological SCD plasmas lead to a specific adhesion property to TSP, in the physiological adhesion test, when compared to the non-incubated counterparts. Almost no adhesion to TSP was observed for the non-incubated AA RBCs freshly sampled from blood units. In parallel, as with the 0.5, 2 or 5 Dyn shear stress values, the mean adhesion results % obtained for SCD plasmas were always significantly higher than for the “HD” plasmas from healthy donors. Nevertheless, the largest difference between “HD” plasma and SCD plasma used in the test was obtained for the 5 Dyn adhesion results (Figure 3A, right panel). Finally, the adhesion results obtained for SCD “basal” or “acute” plasmas were not significantly different, in opposition with the previous data about the senescence markers, and notably for PS-exposure.

### 3.4. The Strength of Senescent RBC’ Adhesion to Thrombospondin Is Related to the Clinical Status of the Patient from Whom the Plasma Sample Was Collected

We also investigated the adhesion properties of senescent AA RBCs to TSP by determining the mean RBCs that continue to adhere to TSP at increasing shear stresses, estimating their strength of adhesion (Figure 3B).

We showed that the RBC senescence induced by plasma samples from SCD patients—particularly those collected during an acute phase of the disease, but also those collected at steady state—induced stronger RBC adhesion to TSP when quantified in the physiologic in-flow assay on Vena8 CelliX biochips, than for the plasma samples collected from healthy donors (“HD”). Numbers in percentage indicate on the graphs the respective changes obtained for each category of plasma between the 0.5 Dyn and the 5 Dyn shear stress applied in the test.

### 3.5. Adhesion to Thrombospondin of AA RBCs, Rendered Senescent by SCD Acute Plasma Samples, Is Not Reversed by an Annexin V Pretreatment

**Figure 4:**
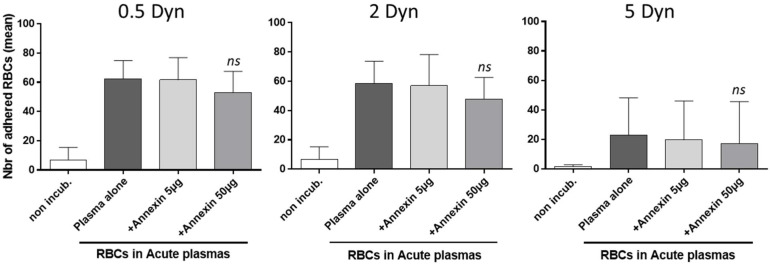
Adhesion results for AA RBCs to thrombospondin, whose senescence is induced by acute SCD plasmas, is not blocked by Annexin V. The adhesion of AA RBCs from blood units was tested under physiological in-flow conditions after co-incubations with plasmas from SCD patients into severe “acute” clinical conditions, using Vena8 microslides coated with TSP. RBCs were washed and resuspended in the Ca^2+^ containing buffer from the kit, before processing the adhesion assay. Recombinant Annexin V (5 and 50 µg) was then added before perfusion to inhibit adhesion. We previously verified that the addition of recombinant Annexin V (5–10 µg) to fresh normal RBCs was sufficient for blocking the positive staining with Annexin V-FITC of PS-positive AA RBCs treated with the calcium ionophore A23187 (data not shown). After washouts at increasing shear stresses (first 0.5, then 2 and finally 5 Dyn/cm^2^), adherent red cells were counted. The means of adhesion for non-incubated RBCs were 6.8 ± 8.6 for the 0.5 Dyn shear stress, 6.7 ± 8.5 (2 Dyn) and 1.6 ± 1.2 (5 Dyn), respectively. For the RBCs incubated with plasmas alone, results were 62.4 ± 12.4 (0.5 Dyn), 58.5 ± 15.1 (2 Dyn) and 23.1 ± 25.1 (5 Dyn), respectively. These results for adherence were not significantly different to the adherence obtained for the condition + Annexin 50 µg; 52.9 ± 14.9 (0.5 Dyn), 47.8 ± 14.8 (2 Dyn) and 17.1 ± 28.5 (5 Dyn), respectively. Results represent the mean of three independent experiments. Data represent the mean of three to five independent experiments. *ns*: non-significantly different to the “plasma alone” condition (ratio-paired *t* test).

If PS exposure on senescent AA RBCs is mechanistically involved in the in-flow adhesion of those particular RBCs to TSP, then the blockade of PS with Annexin V should significantly decrease the level of adhesion observed in adhesion assays to TSP (Figure 4). Previously, we verified in preliminary tests that the addition of recombinant Annexin V (5 or 10 µg) to fresh normal AA RBCs from units, was sufficient to block the positive staining with Annexin V-FITC of RBCs rendered positive for PS by treatment with the calcium ionophore A23187 (data not shown). Here we found that the addition of recombinant Annexin V (5 and 50 µg) to diluted AA RBCs, rendered senescent by SCD acute plasma, had no significant effect on the number of adherent RBCs, regardless of the shear stress considered (0.5, 2 or 5 Dyn/cm^2^, respectively).

### 3.6. The Adhesion of Senescent RBCs to Thrombospondin Can Be Reproduced In Vitro with an Agonist of Piezo1 and Blocked by Gardos Channel Inhibitors

Calcium influx into red cells is a central event that lead to the RBC senescence mechanism. Furthermore, the stimulation of calcium influx by acting on RBC channels with a calcium ionophore can rapidly modify the adhesion properties of normal RBCs to vascular vessel wall proteins [17,30].

**Figure 5:**
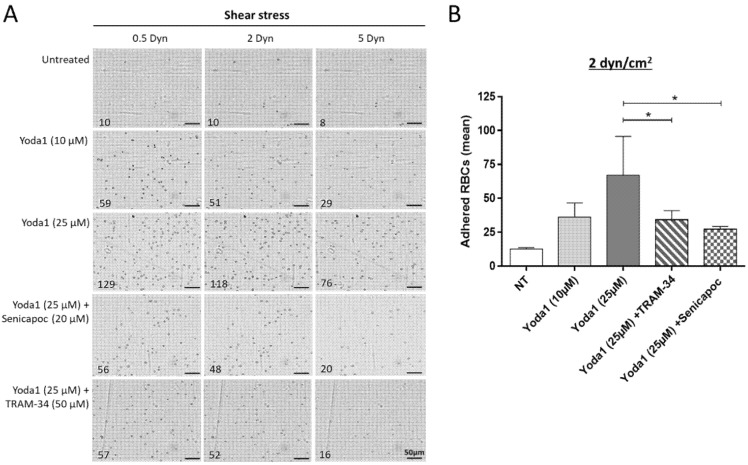
Adhesion of senescent AA RBCs to thrombospondin in vitro mimicked by chemical modulators of Piezo1 and inhibitors of the Gardos channels. Adhesion results for native AA RBCs from blood units and tested under physiological in-flow conditions using the Vena8 microslides coated with thrombospondin (TSP). Treatments were made with Yoda1 (10 and 25 µM) alone or in combination with the inhibitors of the Gardos channel, TRAM-34 (50 µM) or Senicapoc (20 µM) before perfusion. Adherent RBCs were counted in five representative areas at the three consecutive increasing shear stresses (first 0.5, then 2 and finally 5 Dyn/cm^2^). (**A**) A representative photograph of RBCs adhered is shown for each condition, with the mean numbers of RBCs adhered indicated for each shear stress applied. The scale corresponds to 50 µm. (**B**) Results represented on the histograms show the adhesion mean results corresponding to a shear stress applied of 2 Dyn/cm^2^. The means of RBC adhesion results were: 12.6 ± 1.1 for non-treated RBCs (white bar); 36.2 ± 10.5 (light gray bar) and 66.9 ± 28.3 (dark gray bar) for the conditions treated with Yoda1 (10 and 25 µM, respectively); and 34.2 ± 6.7 (hatched bar) and 27.2 ± 2.1 (dotted bar) for the conditions treated with Yoda1 with the inhibitors TRAM-34 and Senicapoc, respectively. Data represent the mean of three independent experiments with * *p* < 0.05 (ratio-paired *t* test).

In parallel, dense dehydrated RBCs observed in SCD patients have the ability to adhere to vessel wall proteins [16]. Considering all these facts, we wanted to determine whether the observed adhesion of RBCs after plasma challenge can be reproduced in vitro. To test this, normal AA RBCs from units were left untreated or treated with the Piezo1/TRPC5 activator Yoda1 and assayed for in-flow adhesion in biochips coated with TSP (Figure 5).

When native AA RBCs from units showed only slight adhesion to TSP (12.6 ± 1.1%), the Yoda1 treatment (10 and 25 µM) induced a dose-dependent increase in RBC adhesion to TSP in that physiologic assay, up to 66.9 ± 28.3%. When RBCs stimulated to adhere with Yoda1 (25 µM) were pretreated with the specific inhibitors of the Gardos/KCa3.1 channel (Senicapoc and TRAM-34), their adhesion to TSP decreased to reach the values of 34.2 ± 6.7 and 27.2 ± 2.1%, respectively. Such a result clearly shows that abnormal activation of Piezo1 and Gardos ion channels in RBC membranes can modulate the adhesion of normal RBCs to the vascular wall-associated proteins.

## 4. Discussion

We confirmed here that pathological plasma samples from SCD patients can directly affect ex vivo the integrity of (HbA) RBCs from blood units, when in contact with them. The deleterious impact of plasmas can alter several properties of RBC membranes, including cation permeability (calcium influx), dehydration (cell shrinkage or reduction in RBC size) and phospholipid scrambling (exposure of PS on the outer membrane)

Previous findings reported by Lang et al. [21,22,31] or Kempe et al. [32] demonstrated the deleterious impact of acute plasmas. In our laboratory as well as already published works with plasma from acute-phase SCD patients, or from those experiencing delayed hemolytic transfusion reactions [9,23], or from other pathological contexts [12] reported a similar impact on normal AA red cells leading to accelerated senescence. However, none of these studies were obtained on such a large number of individual patient samples (*n* = 54) as we describe here.

From a mechanistic point of view, we concluded that the strong proinflammatory environment found in plasmas cannot by itself promote rapid alterations to RBCs [12]. Instead, soluble plasma factors, other than inflammatory cytokines and present in SCD plasma samples, may be responsible for accelerated senescence.

One of the key features demonstrated here is the relationship existing between the markers of RBC senescence. The three markers quantified after challenge with plasma samples (RBC size decrease, PS exposure and calcium influx), that characterize senescence, were found to be closely correlated after a T 48 h plasma contact. Although this result was not unexpected, it has never been demonstrated before. Our results particularly focused on the phospholipid PS easily detected on senescent AA RBCs.

PS-exposure can provoke the destruction of immunoglobulin-sensitized RBCs by macrophages or activated complement [26,33]; it may also increase RBC adhesion to vessel walls [20,24,34], a mechanism directly implicated in VOCs occurring in SCD [35], this particularly when endothelial cells are activated due to oxidative stress in severe contexts [36,37]. In previous works from our team, we showed that PS-exposure on transfused AA RBCs, as well on the SS RBCs of the recipient patient with SCD, can be a major factor in the potentiation of the hemolytic transfusion reaction [9]. PS-exposing red cells may also be hydrolyzed by secretory phospholipase A2 (sPLA2), a plasmatic factor produced in SCD patients during acute chest syndromes. This mechanism, by generating lysophosphatidic acid, may result in vascular dysfunction [36,37,38], another deleterious consequence of transfusion in SCD.

Instead of being impacted by sPLA2, we rather imagine that free Hb or free heme byproducts of RBC hemolysis, present in the SCD plasma samples used in co-incubation experiments, are the factors that may provoke the senescence of AA RBCs, through oxidative stress, in our model. Gatidis et al. [11] reported that hemin induce suicidal RBC death, and Lang et al. [39] observed that RBCs incubated with plasma from patients with hemolytic uremic syndrome exhibit increased PS-exposure. The SCD plasma samples tested here contain free Hb (although these levels were not specifically quantified for the study), especially in the plasmas obtained from patients into severe phases of the disease (VOC or ACS episodes). The byproducts of previous RBC destruction may be an important component of the complications of the disease through leading to red cell damage, in addition to their implication in endothelial dysfunction. We showed here that the changes occurring in RBC hydration, through direct effects on membrane channels, can play a role on RBC adhesion to endothelium and further removal from the circulation. We are not the ones to study the relative implication of dehydration or ageing on the fate of RBCs in circulation or after transfusion (see also: Supplementary Figure S4).

Recent works from Klei et al. on Gardos and Piezo1 relative activities on RBC removal, by way of acting on RBC dehydration and deformability, have also shed new insights [15,40] on this mechanism. Rogers and Lew also recently described the Ca^2+^-dependent K+ efflux erythrocyte senescence driven by intracellular Ca2+ activating the Gardos channel. In their context, the mechanism is also due to Piezo1-mediated calcium permeation, a phenomenon that can be described on red cells physiologically aged in circulation, on pathological sickle erythrocytes, or RBCs extensively stored for transfusion or artificially dehydrated [41]. Wadud et al. also suggested that the use of Yoda1 as a powerful Piezo1 agonist is an easy way to investigate its role in red cell senescence due to calcium influx [42].

In parallel to the implication of Piezo1 we tested in that study, the mechanistic insights about calcium influx-related sickle RBC dehydration are much larger and more multifactorial [43]. Calcium influx in red cells may be multi-faceted in SCD and not only regulated by Piezo1 (although, here in this work, we were interested in only donor AA RBCs). First, the Piezo1 way is not the only or primary pathway for RBC dehydration, as Piezo1 is a mechanosensitive membrane channel. Secondly, pharmacologically inhibiting calcium-dependent protease Calpain, abundantly present in RBCs, reduces sickle RBC dehydration and Gardos-channel activity [44]; genetic ablation of calpain also showed reduced pain in sickle mouse [45].

The thrombospondin (TSP) vessel matrix protein was chosen here as the coated molecule into Vena8 biochips for in-flow adhesion tests. We previously verified that senescent RBCs after SCD plasma contact did not lead to adhesion with the two most common extracellular matrix (ECM) proteins Laminin or Fibronectin [28,46] (data not shown). TSP can create multimolecular bridges between RBCs and endothelial cells or ECM, and it can promote or help red cell adhesion [47]. Furthermore, TSP levels have also been reported to be increased during acute events such as VOC and associated with the occurrence of ACS episodes in SCD [48]. Effectively, in our conditions, we detected a strong adhesion of donor AA RBCs to TSP, following incubation with SCD plasmas in acute episodes.

Depending on the bridging molecule considered to be involved in adhesion to the vessel wall (either TSP, Laminin or Fibronectin) [28], the molecular partners participating in the adhesion mechanism would be PS, α4β1, CD36 or CD47/IAP, respectively. First, we can rule out a role for CD36/GPIIIb, as CD36-negative RBCs can adhere to TSP [49,50]; integrin α4β1 is expressed only on reticulocytes and would not therefore be present on mature donor RBCs in blood bags [39]. Therefore, we focused on PS and the impact of calcium concentration on the RBC adhesion mechanism to TSP. Nevertheless, incubating recombinant Annexin V with senescent RBCs before the flowing assay had no significant effect on adhesion, in our conditions Therefore, CD47/IAP was also a candidate protein that may influence the RBC adhesion to TSP that we detected.

The TSP–CD47 signaling pathway can mediate RBC survival and deformability by in mediating Ca^2+^ influx into red cells [51], and a TSP treatment can generate reactive oxygen species ROS in pulmonary artery endothelial cells, which is abrogated by CD47 blockade. TSP and CD47 were upregulated in SCD patients with pulmonary hypertension (PH) in their conditions and they hypothesized the TSP–CD47 signaling promotes PH, in part, by increasing reactive oxygen species (ROS) [52]. Here in our work, we were not able to detect any changes in the level of CD47 on AA RBCs after plasma challenge, nor did we quantify any effect of the anti-CD47 blocking antibody (clone 2D3; specific for the oxidized form of CD47: oxCD47) on senescent RBCs. In parallel, the detection of the oxCD47 was only weak in our conditions and was not further described in the study.

## 5. Study Limitations

We showed here that red cells from units can suffer stress after exposure to plasma samples from SCD patients. Nevertheless, some limitations in the study and our results can be pointed out.

One fact that limits the study’s originality is that we are not the first to describe this ex vivo deleterious effect of pathological plasma obtained from patients on RBCs from units, as reported by Lang et al. or Kempe et al.; also in our laboratory, we already described that the acute plasmas from various pathological contexts can have such an impact on red cells leading to senescence.

Another limitation, in the way to definitively prove the mechanistic scenario of calcium-induced adhesion, could be the absence of control experiments in the adhesion tests with the sickle plasma-treated AA-RBCs, pre-treated or not with Gardos inhibitors. Furthermore, we need also to increase the number of plasma samples from SCD patients (i) to implement results concerning the in-flow physiological adhesion tests in biochips and (ii) the in vitro effects of chemical effectors of RBC channels on adhesion to upgrade the significance of our results.

In the experiments with recombinant Annexin V, a bias could be that Annexin V added did not totally bind to the large amount of PS-exposed on senescent RBCs. In further experiments, it could be important to verify this fact with rapid FACS analysis of PS-positive senescent RBCs pre-incubated with similar amounts of Annexin V.

Calcium influx in RBCs can be considered multi-faceted, as it is not only regulated by the only Piezo1 channel, with TSP and CD47 directly implicated in it. The fact that the TSP–CD47 signaling pathway wasn’t explored in our work could be considered as a weak point.

Finally, further biochemical studies will be required now to clearly identify the mediators in the plasma that potentiate the transfusion-driven alterations to AA RBCs.

## 6. Conclusions

We showed that AA red cells from blood units can suffer stress after exposure to plasma samples from SCD patients. Nevertheless, at this time point, how TSP and senescent AA RBC interacts is yet to be determined. It will be now of importance to better understand the in vivo fate of transfused RBCs in such pathological contexts, collecting and testing a higher number of SCD samples, as this phenomenon can have adverse clinical consequences for the patients.

We think that new insights on RBC senescence and their connection to red cell adhesion to the vessels, will give an easy way to overcome, in the future, the deleterious occlusive events in SCD. Clinical studies might be necessary to confirm these results obtained ex vivo along with mechanistic studies on the byproducts of RBC destruction, factors which may play a role in the RBC damage. How to overcome senescence may now be the key to discovering successful future treatments of anemia or hemolytic transfusion reactions in SCD. The capability we demonstrated here, to easily modulate the adhesion of AA RBCs to vascular-wall proteins, might represent a promising target for the treatment of occlusive events in that disease.

## Data Availability

All the obtained data used to support the findings of this study are available from the corresponding author upon reasonable request.

## Supplementary Materials

The following are available online at https://www.mdpi.com/article/10.3390/biom11070963/s1, Figure S1: Dot plot and histogram FACS representations of the PS-exposure parameter after incubations of AA RBCs with sickle-cell patient plasma, Figure S2: Factorial analysis with two-way ANOVA revealed that the clinical state of the patients’ plasmas tested (“steady state” versus “acute phase”) had a highly significant effect (*p* < 0001) on the PS-exposure senescence parameter, Figure S3: Scheme representing the different situations that influence the red blood cell fate in various conditions affecting ion channels described in the study, Table S1. Results for the two-way ANOVA factorial analysis exploring the effects of the clinical state of the patients’ plasmas tested (“steady state” versus “acute phase”) and duration of the RBC/plasma contact (T 24 h and T 48 h), and the interaction between these two variables, on a PS-exposure parameter.

## Abbreviations

RBCs: red blood cells; SCD: sickle-cell disease; MFI: mean fluorescence intensity; MCHC: mean corpuscular hemoglobin concentration; Hb: hemoglobin; PS: phosphatidylserine; VOC: vaso-occlusive crisis; ACS: acute chest syndrome; Hct: hematocrit; FITC: fluorescein-isothiocyanate; DMSO: dimethylsulfoxide; sPLA2: secretory phospholipase A2; a.u.: arbitrary units.
