# Supplementary material for: Ex Vivo Activation of Red Blood Cell Senescence by Plasma from Sickle-Cell Disease Patients: Correlation between Markers and Adhesion Consequences during Acute Disease Events
Source: Biomolecules. 2021 Jun 30;11(7):963. doi: 10.3390/biom11070963 (PMC8301992; doi:10.3390/biom11070963)
Supplement: Supplementary file 1 [file biomolecules-11-00963-s001.zip › biomolecules-1245928-supplementary.pdf]

Supplementary Material

1. Supplementary Figures

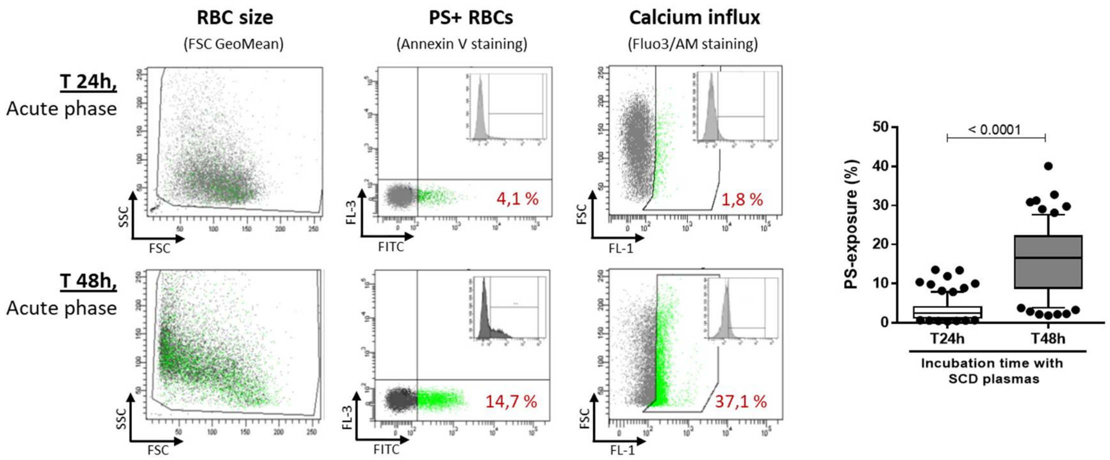

Supplementary Figure S1. Dot plot and histogram FACS representations of the PS-exposure parameter after incubations of AA RBCs with sickle-cell patient plasma. The AA RBC units' blood groups and those of the patients giving the plasmas were phenotypically matched for co-incubations. Phosphatidylserine (PS) externalization results obtained for the 24-hour (T 24 h) and 48-hour (T 48 h) incubations at +37 °C with sickle-cell disease (SCD) plasma samples are represented on the same graph, considering the clinical condition ("steady state" versus "acute phase") (see also: Supplementary Table S1). A box and whisker plot (on the right) representing the time-dependent PS-exposure % quantification on RBCs is shown, after incubations with sickle plasmas without considering the clinical condition of the patients. The results obtained were 3.34 ± 2.9 % at T 24 h and 16.04 ± 8.7 % at T 48 h ($p < 0.0001$; Wilcoxon matched-pair test).

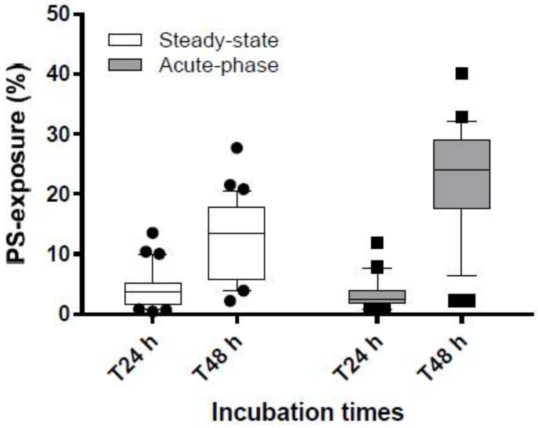

Supplementary Figure S2. Factorial analysis with two-way ANOVA revealed that the clinical state of the patients' plasmas tested ("steady state" versus "acute phase") had a highly significant effect (P < 0001) on the PS-exposure senescence parameter. The duration of the RBC/plasma contact (T 24 h and T 48 h) had a less but also significant effect (P = 0001); a significant interaction between the two variables is observed (P < 0001)

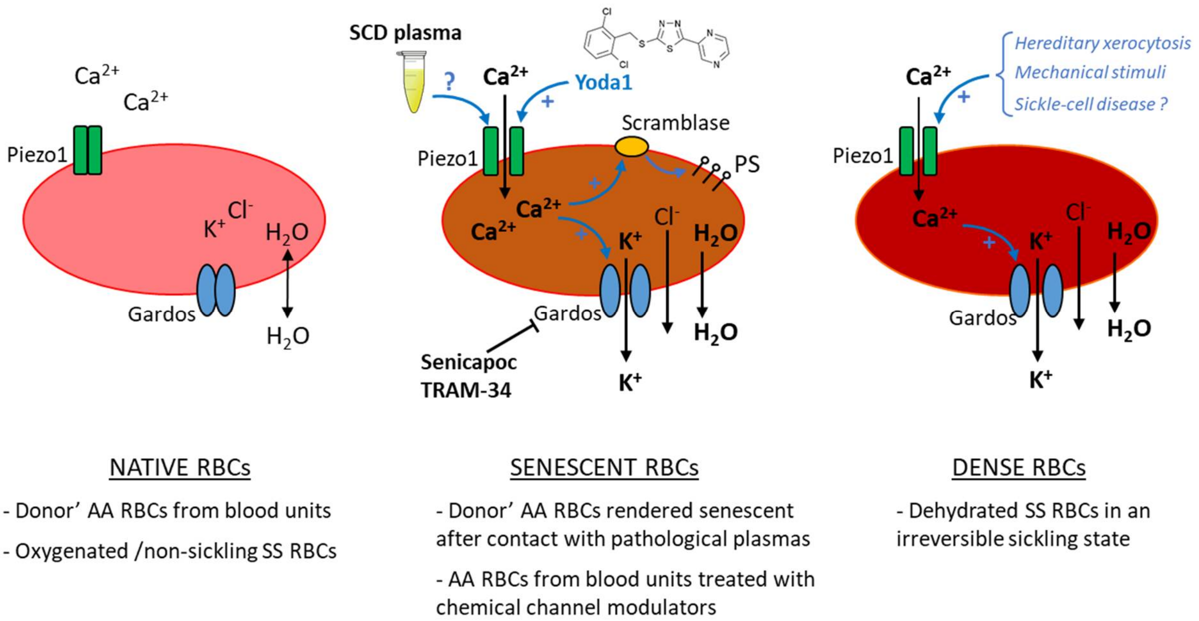

Supplementary Figure S3. Scheme representing the different situations that influence the red blood cell fate in various conditions affecting ion channels described in the study. (**Left**) In native functional red cells, Piezo1 and Gardos channels are closed; an equilibrium in the H_2O concentrations inside and outside the cells maintain the RBCs in optimal shape and size. (**Center**) Opening of the Piezo1 channels (green) by the molecule Yoda1 promotes an influx of calcium ions, stimulating the opening of the Gardos channels (blue). Unknown soluble factors, present in plasma samples obtained from SCD patients, also have a similar ability to that described in the present study. The resulting efflux of potassium ions through the KCa3.1/Gardos channel leads to a loss of water from the red cells, generating senescent RBCs, characterized by markers of senescence exposed and cell shrinkage. (**Right**) Excessive opening of the Piezo1 channel might be involved either by mechanical stress or when mutations in the *piezo1* gene reduce its ability to close, which causes RBCs to shrink in *xerocytosis*. Mechanisms in sickle-cell disease might exist, acting on functional Piezo1 channels, that lead to the formation of dense (dehydrated) RBCs. Modified from Patel A. et al. and Danielczok J.G. et al. [1,2].

Supplementary Table S1. Results for the two-way ANOVA factorial analysis exploring the effects of the clinical state of the patients' plasmas tested ("steady state" versus "acute phase") and duration of the RBC/plasma contact (T 24 h and T 48 h), and the interaction between these two variables, on a PS-exposure parameter. The clinical state of the patients' plasmas tested had a high significant effect ("SCD state", $P < 0001$) on the PS externalization on red cells. The duration of the RBC/plasma contact had a less but also significant effect ("incubation Time", $P = 0001$) and a significant interaction between the two variables is observed ($P < 0001$).

Supplemental table S1: Two-way ANOVA analysis made on PS-exposure (%) senescence parameter

Two-way ANOVA:	Ordinary
Alpha:	0,05

Source of Variation	% of total variation	P value	P value summary	Significant?
Interaction	8,173	< 0,0001	****	Yes
'SCD State' factor	52,05	< 0,0001	****	Yes
'Incubation Time' factor	5,706	0,0001	***	Yes

ANOVA table	SS	DF	MS	F (DFn, DFd)	P value
Interaction	784,0	1	784,0	F (1, 104) = 22,45	P < 0,0001
'SCD State' factor	4994	1	4994	F (1, 104) = 143,0	P < 0,0001
'Incubation Time' factor	547,4	1	547,4	F (1, 104) = 15,67	P = 0,0001

2. Supplementary Methods

2.1. Study Design

2.1.1. Patients and Donors:

Plasma samples were obtained from 54 randomly selected adult patients with a definitive diagnosis of SCD (homozygous Hb SS) from the Henri Mondor Hospital (Créteil, France). We verified that none of the patients presented either a Sβ0, Sβ1, or SC phenotype associated with SCD. Patients were followed for routine explorations outside of painful episodes (steady state; n = 34) or during hospitalization for acute symptoms (acute phase; n = 20). Acute symptoms were vaso-occlusive crisis (VOC, defined as pain or tenderness affecting at least one part of the body and requiring opioids), associated or not associated with acute chest syndrome (ACS, diagnosed in the presence of an associated respiratory symptom (dyspnea or chest pain)) [26]. In parallel, and where indicated, healthy volunteers were included and tested as a control population (HD). The blood groups of the RBC units and of the patients supplying plasma were selected to ensure phenotypic matching. Cross-making was also performed, to ensure compatibility, as previously described [9].

2.1.2. Packed RBC Units, Storage and Sampling:

Fresh leucoreduced RBC units were obtained from the EFS Preparation Service (Rungis, France), removed from the therapeutic delivery circuit, and received by our laboratory on day 3 post-collection. Briefly, fresh donated bloods from healthy donors were subjected to centrifugation, then to leukofiltration and the RBCs obtained were transferred into concentrate bags, and stored in saline-adenine-glucose-mannitol solution (SAGM) at +2 to 6 °C in a standard blood bank refrigerator until day 42. Small RBC fractions were then aseptically removed from units; they were washed three times in 0.9 % NaCl and processed immediately.

2.1.3. Patients' Sample Collection and Preparation:

Peripheral venous blood samples from patients and donors were collected in ethylene-diamine-tetra acetate (EDTA) tubes and processed within the first 24 h. Biological parameters and blood cell counts were determined using a Micros ES60 counter (Horiba ABX, Montpellier, France). Plasma samples were isolated after centrifugation at 2880 g for 10 min, aliquoted and frozen immediately at –80 °C.

2.1.4. Ex Vivo Co-Incubations of RBCs with Plasma:

Selected plasma samples from SCD patients or healthy donors were thawed and incubated with AA RBCs at an 8 % hematocrit (Hct) drawn from refrigerated stored concentrates. Before, the RBCs were washed twice in 0.9 % NaCl and packed by centrifugation. Plasma samples and AA RBC units were chosen from compatible blood groups. Each plasma sample was tested 2-3 times with different RBC samples from independent units to minimize bias due to inter-individual variability between RBC concentrates. The incubations were performed at +37 °C for 24 and 48 hours as previously described [12,23] and cellular markers of RBC senescence measured. Incubations of less than 24 hours (for 6 and 12 h) did not induce the appearance of any senescence markers, in our conditions.

2.2. RBC Staining and FACS Analysis

Senescent AA RBCs obtained after co-incubations with plasma samples were analyzed by flow cytometry. 100 µL aliquots of a 0.2 % Hct suspension were washed in PBS, packed by centrifugation and suspended in the specific saline buffered solution before analysis on a FACS Canto 2 flow cytometer (BD, Franklin Lakes, NJ, USA) using the DIVA 6.0 software. To suppress non-RBC-associated events, populations were defined by gating a region on the forward- and side-scatter plots. 10,000 events were analyzed per run.

2.2.1. Phosphatidylserine-Exposure and Forward Scatter Determinations

The RBCs were tested for phosphatidylserine (PS)-exposure by flow cytometry, based on the detection of Annexin V-positive cells with the APOPTEST fluorescein-isothiocyanate (FITC) kit, as described by the manufacturer. The PS-exposure threshold for a positive reading was 0.2 %. In parallel, the forward scatter (FSC) value, as a measure of the size and volume of the cells, was determined using unstained RBCs [12,23].

2.2.2. Measurement of Intracellular Calcium Levels

To determine changes of intracellular calcium levels attesting Ca^{2+} influx, RBCs were washed in HBSS and loaded with the cell-permeable molecule Fluo3/AM (2 µM) in HBSS containing 2 mM $CaCl_2$ (HBSS/$CaCl_2$) for 20 min at 37 °C. The Fluo3/AM-loaded RBCs were then washed twice, suspended in HBSS/$CaCl_2$ and the fluorescence was measured at FL-1 (emission wavelength 506-526 nm). Results are represented as the percentage of Fluo3/AM positive RBCs. Data collected in parallel as the mfi of Fluo3/AM-stained RBCs gave similar results.

2.3. In-Flow RBC Adhesion Assays in Microslides

The flowing adhesion of senescent AA RBCs (incubated for 48 hours with sickle plasmas) to thrombospondin (TSP) was measured under physiological flowing conditions, as previously described [27,28]. First, TSP from human platelets (10 µg/mL) was immobilized in Vena8 microslides (Cellix Ltd, Dublin, Ireland) by incubation overnight at +4°C. The senescent AA RBCs obtained after co-incubations with the plasma samples were washed and resuspended in Hank's buffer supplemented with 0.2 % BSA. The RBC suspensions obtained (hematocrit 0.5 %) were perfused through the microslide with VenaFlux Assay 2.3 analysis software, on a fully automated Z1 microscope (Carl Zeiss, Obertochen, Germany), at a shear stress of 0.5 Dyn/cm^2 for 5 min at +20 °C. Washouts were then performed with Hank's buffer, at increasing shear stresses from 0.5, then to 2 and finally to 5 Dyn/cm^2, for 5 min each, at +20 °C. After washing, adherent cells were counted in five representative areas along the centerline of the microslide, with the ImageJ software. Where indicated, the implication of PS-exposure in the senescent RBC adhesion process to TSP was tested by the addition of recombinant Annexin V (5 and 50 µg) to diluted AA RBCs in 2 mL of HBSS /BSA (corresponding to final concentrations of Annexin V: 2.5 µg and 25 µg/mL) for 10 min. incubation at +4 °C before processing to flowing adhesion assays. All the experiments using Annexin V were made in HBSS buffer with calcium and magnesium to allow it to bind.

Before flowing adhesion assay, native AA RBCs were treated with the Piezo1/TRPC5 channel agonist Yoda-1 (50 mM stock in DMSO; Sigma-Aldrich, St. Louis, MI, USA) and the Gardos/KCa3.1 inhibitors Senicapoc (10 mM stock; Clinisciences, Nanterre, France) or TRAM-34 (10 mM stock; R&D Systems, Minneapolis, MN, USA), if required. Suspensions of washed AA RBCs (0.5 % Hct) from blood units were treated for 30 min at +37 °C with or without Yoda-1. Where indicated, the suspensions were subjected to pretreatments by incubation for 10 min with one of the indicated channel inhibitors. Suspensions of treated RBCs (0.5 % Hct) were then placed on ice and immediately tested for in-flow adhesion.

2.4. Solutions and Chemicals

Phosphate buffered saline (PBS) and Hank's balanced salt solution (HBSS) were obtained from Invitrogen. The saline-adenine-glucose-mannitol (SAGM) solution (150 mmol/L NaCl, 1.25 mmol/L adenine, 50 mmol/L glucose, 29 mmol/L mannitol, pH5.6) was obtained from Fresenius (Fresenius Kabi, The Netherlands). The Apoptest fluorescein-isothiocyanate (FITC) kit was obtained from Dako (Trappes, France). The Ca^{2+} dye Fluo-3/AM was obtained from Calbiochem (Bad Soden, Germany). Thrombospondin from human platelets (TSP, 10 µg/mL) was from Merck Millipore (Darmstadt, Germany). The Piezo1/TRPC5 channel agonist Yoda-1 was from Sigma-Aldrich (St. Louis, MI, USA); the Gardos/KCa3.1 channel inhibitor Senicapoc was obtained from Clinisciences (Nanterre, France) and TRAM-34 from R&D Systems (Minneapolis, MN, USA),

2.5. Statistics

Quantitative data are expressed as the arithmetic mean ± SD and categorical data as numbers with percentages. In vitro treatments and adhesion experiment as well as ex vivo co-incubations RBCs /plasmas were independently repeated at least three times. Statistical analysis was performed using 2-tailed t tests (Wilcoxon matched-pairs rank test or Mann–Whitney unpaired test, as appropriate); analyses of variance were carried out with Kruskal–Wallis test or ANOVA, as appropriate.

A two-way ANOVA factorial analysis was carried out to find out the respective effects and interactions of the times of incubation (T24 h versus T48 h) and the types of plasma ("steady state" versus "acute phase") on the PS-exposure RBC parameter. The statistical software program GraphPad Prism version 6.01 (GraphPad Inc, La Jolla, CA, USA) was used for statistical analyses. Values of $p < 0.05$ were considered statistically significant. Linear regressions and correlations were also made to test the relationship between the senescence markers for the patients' plasma tested for whom we collected results for the three different markers (n = 20) using the Stata software (StataCorp LLC, College Station, TX, USA); both Pearson and Spearman correlation tests were used to determine the relationship between 2 variables.

3. Supplementary References

1. Patel, A.; Demolombe, S.; Honoré, E. An alternative to force. *Elife* **2015**, *4*, e08659.
2. Danielczok, J.G.; Terriac, E.; Hertz, L.; Petkova-Kirova, P.; Lautenschläger, F.; Laschke, MW.; Kaestner, L. Red Blood Cell Passage of Small Capillaries

Is Associated with Transient Ca2+-mediated Adaptations. Front. Physiol. 2017, 8, 979.
